# Fostering organisational commitment: a resilience framework for private-sector organisations in South Africa

**DOI:** 10.3389/fpsyg.2024.1303866

**Published:** 2024-02-15

**Authors:** Ester Mujajati, Nadia Ferreira, Melissa du Plessis

**Affiliations:** Department of Human Resource Management, University of South Africa, Pretoria, South Africa

**Keywords:** organisational commitment, resilience, career adaptability, job embeddedness, hardiness, talent retention, private sector organisations

## Abstract

**Introduction:**

Organisations worldwide encounter three significant and challenging issues related to talent management: intense competition for skilled employees, elevated rates of employee attrition, and the ongoing struggle to attract top-tier talent. This research focused on investigating the interconnected dynamics among factors associated with employee retention, including organisational commitment, job embeddedness, and hardiness, in conjunction with resilience-related behaviours such as resilience and career adaptability.

**Methods:**

A cross-sectional survey design was used to gather quantitative data from a convenience sample of employees within the private sector in South Africa (*N* = 293). The self-assessments of the participants were assessed using a range of well-established and validated instruments. Correlation and regression analyses, followed by structural equation modelling, were utilised to construct a resilience framework designed specifically for private sector organisations in South Africa.

**Results:**

The results reveal significant associations between organisational commitment, job embeddedness, and hardiness (as retention-related attributes) and resilience and career adaptability (as resilience-related behavioural capacities). These relationships served as the basis for the development of a resilience framework for employees in South African private organisations.

**Discussion:**

In South African private-sector organisations, talent retention is crucial due to a talent shortage. The study found that employees have a strong emotional attachment to their organisations, are highly aligned with their jobs and communities, and display resilience. Organisational commitment, job embeddedness, and hardiness are key factors in reducing turnover, forming an effective retention strategy. This research contributes to the development of a resilience framework for South African private sector organisations.

## Introduction

Considering the ongoing global talent shortage, organisations are increasingly seeking effective strategies to retain their top-performing employees. These individuals are essential for driving organisational performance and maintaining a competitive edge (Zhang et al., 2022; Association of Chartered Certified Accountants (ACCA), 2023; Durth et al., 2023). Unfortunately, the African continent faces significant challenges in retaining its highly skilled workforce due to issues like inadequate compensation, unattractive work environments, socio-economic challenges, and political instability (Ebeye and Lee, 2023; Ombogo, 2023). Moreover, high employee turnover rates continue to pose a significant and persistent challenge for South African private sector organisations, necessitating a focused effort on talent retention (Musakuro and De Klerk, 2021; Maloba and Pillay-Naidoo, 2022; Snyman et al., 2022; Tongchao et al., 2022).

Recent research findings have shed light on the multifaceted reasons that lead employees to consider leaving their workplaces. These reasons encompass various elements, including job stress, levels of job dissatisfaction, perceptions of job security, the quality of the work environment, motivational factors, and the adequacy of wages and rewards (Al-Suraihi et al., 2021). In the context of South African private sector organisations, a persistent issue of high employee turnover rates has been linked to factors such as unfavourable work environments, employee dissatisfaction with their roles, and a deficiency of both intrinsic and extrinsic motivation (Maloba and Pillay-Naidoo, 2022).

Furthermore, results from regression analysis conducted by Snyman (2022) have highlighted the interconnected nature of psychological contract factors, including employer obligations, employee obligations, job satisfaction, and the state of the psychological contract. These psychological contract factors have demonstrated significant associations with critical employee retention elements such as compensation, job characteristics, training and development opportunities, supervisor support, career advancement prospects, and work-life balance. Retaining top talent is not merely a desire but a strategic imperative for private sector organisations as it directly influences their ability to achieve sustainable growth and maintain competitiveness (Ebrahim et al., 2021).

In addressing the pressing issue of high employee turnover rates in South African private sector organisations, a comprehensive range of strategies has been employed. These strategies encompass elements aimed at creating a more conducive and appealing work environment to increase employee retention. These elements include equitable compensation packages, the cultivation of favourable work environments, the provision of attractive benefits, the implementation of incentives to recognise and reward high performance, career development, and the extension of essential support systems (Snyman, 2022; Swanepoel and Saurombe, 2022).

However, retaining top talent is far from one-dimensional. It extends beyond the conventional realms of compensation and rewards. Instead, it encompasses the critical dimensions of creating a satisfied work environment and effectively understanding and addressing employee expectations (Ngobeni et al., 2022). Achieving employee satisfaction and ensuring alignment between their expectations and organisational objectives have emerged as fundamental aspects of the talent retention equation. Moreover, talent retention is not solely about job satisfaction; it is also about retaining and nurturing the knowledge, unique skills, and expertise that employees bring to the organisation. These knowledgeable individuals serve as vital contributors to an organisation’s competitiveness and overall success (Mantje et al., 2023). Therefore, retaining them is paramount in the highly competitive South African private sector.

To address these challenges and maintain competitiveness, South African private sector organisations must move beyond traditional retention strategies. They must invest in fostering retention-related dispositions such as organisational commitment, job embeddedness, and hardiness, as well as resilience-related behavioural capacities like resilience and career adaptability. Understanding the interplay between these factors is vital in developing strategies that enable organisations to effectively retain their talented employees in a dynamic and competitive landscape.

While numerous studies have explored the fierce competition for talented employees, high turnover rates, and the global war for talent (Wolfswinkel and Enslin, 2020; Amushila and Bussin, 2021; De Smet et al., 2022; Mazlan and Jambulingam, 2023), there is a notable gap in research specific to South African private sector organisations. This gap pertains to the investigation of the relationship dynamics between retention-related dispositions and resilience-related behavioural capacities, and how these factors can aid private-sector organisations in retaining their talented employees.

Furthermore, there is a lack of in-depth understanding regarding the psychological aspects that contribute to employee retention, which this research aims to address. Additionally, it appears that existing retention models may not be sufficiently effective in the ongoing “war” for talent. This research article seeks to fill these critical gaps and provide valuable insights into talent retention in the South African private sector.

## Theory

### Organisational commitment

Meyer and Allen (1991) contributed significantly to the study of organisational commitment, defining it through three components: affective, calculative, and normative. Affective commitment involves an emotional connection to the organisation, driven by positive emotions from how the organisation treats its employees. Calculative commitment is based on the perceived cost of leaving, often tied to investments in skills and benefits. Normative commitment stems from a sense of duty to stay, influenced by socialisation experiences and beliefs in the organisation’s deserving loyalty. Overall, these three components influence an employee’s decision to stay with the organisation, with affective commitment linked to a strong emotional bond with the organisation (Pieters et al., 2020; Schaap and Olckers, 2020; Els et al., 2021). In addition, committed employees tend to share the organisation’s objectives and values, resulting in increased dedication and extra effort invested in their work. This heightened commitment is positively associated with various positive outcomes, including reduced absenteeism, increased altruism, and conscientiousness, promoting the organisation externally, higher job satisfaction, improved employee well-being, reduced stress, work–family conflict, and heightened motivation (Blersch et al., 2020).

### Job embeddedness

Mitchell et al. (2001) highlighted that job embeddedness stands out as a robust predictor of employee turnover, surpassing conventional attitude variables. It serves as a pivotal factor in shaping employees’ decisions to remain in their roles, bridging the gap between the factors at work and in their personal lives that discourage them from leaving (Wu et al., 2021).

On-the-job embeddedness pertains to an employee’s sense of connection and attachment to their organisation, often referred to as organisational embeddedness. In contrast, off-the-job embeddedness relates to an employee’s ties within a community that anchor them in that context. According to Peltokorpi and Allen (2023), employees with higher levels of on-the-job embeddedness are less likely to entertain thoughts of quitting. Such employees tend to feel invested in their roles and perceive themselves as integral to the organisation, fostering a commitment to remain, even when completely satisfied with their current job. Moreover, those with strong on-the-job embeddedness might perceive fewer alternative job opportunities, reducing their inclination to consider leaving.

Job embeddedness has proven effective in retaining employees both globally and in the South African labour market. For instance, as noted by Potgieter et al. (2018), deeply engaged employees find it increasingly challenging to contemplate leaving their positions and the organisation. Through the lens of person-environment fit theory (Dawis, 1996), job embeddedness reflects an employee’s psychological attachment to valued job characteristics and working conditions, significantly influencing job satisfaction. Strong workplace connections and the alignment of career goals with the organisation reduce the desire to leave, while the prospect of sacrificing perks, career advancement, compensation, and benefits makes departure less appealing (Mitchell et al., 2001; Holtom and O’Neill, 2004; Shibiti et al., 2018; Shibiti, 2019). In summary, higher levels of job embeddedness will foster engagement and ultimately improve employee retention.

### Hardiness

Hardiness, a psychological trait applicable in the workplace, signifies an individual’s capacity to withstand and excel amidst stress, adversity, and challenging situations (Maddi and Kobasa, 1984). It is characterised by three key components, as suggested by Kobasa (1979): commitment, control and challenge.

This trait bears substantial advantages for both employees and organisations. It empowers employees with the resilience to navigate workplace pressures, nurture commitment, maintain control, and perceive challenges as opportunities for growth (Kobasa, 1979; Oral and Karakurt, 2022; Bartone et al., 2023). The outcome is heightened job satisfaction and overall well-being (Lee and Kim, 2023). In the organisational setting, hardiness curbs turnover rates, augments performance in stressful scenarios, and stimulates innovation, thereby enhancing productivity and creativity. Studies propose that individuals possessing heightened levels of hardiness display a reduced propensity for voluntary turnover (Banda, 2019; Marshall and Stephenson, 2020). Their unwavering commitment to their roles and the organisation, coupled with their capacity to not just endure but thrive in challenging circumstances, curtails their inclination to seek alternative opportunities. They tend to view challenges and job dissatisfaction as issues that can be addressed and ameliorated, rather than as grounds for departure.

Ultimately, hardiness plays a pivotal role in talent retention (Coetzee et al., 2018). As mentioned above, those with elevated hardiness are more likely to sustain their commitment to their roles, even when confronted with adversity. Their elevated job satisfaction diminishes the desire to explore other employment options. Furthermore, their resilience and adaptability render them invaluable assets in high-stress environments, where they can consistently deliver effective performance.

### Resilience

Resilience can be defined as the ability to adapt, bounce back, and effectively cope with challenges, setbacks, or adversities, whether they are personal or professional (Southwick et al., 2014). It represents an individual’s capacity to maintain their psychological well-being and performance in the face of stress, pressure, or difficult circumstances.

Resilience holds significant importance for both employees and organisations. For employees, it signifies their ability to withstand the pressures of their roles, maintain a positive outlook, and continue to perform at their best, even when confronted with adversity (Shatté et al., 2017; Ferreira and Gomes, 2021; Hanu and Khumalo, 2023). For organisations, resilience plays a pivotal role in reducing voluntary turnover and mitigating intentions to leave (Hidayah and Ardiansyah, 2019; Hartmann et al., 2022). When employees possess a high level of resilience, they are more capable of managing workplace stressors, job dissatisfaction, or other factors that may trigger a desire to leave (Albalá-Genol et al., 2023). Resilient individuals are better equipped to seek solutions and adapt to changing circumstances rather than resorting to the option of quitting. In essence, resilience acts as a buffer against the negative impact of workplace stress and can discourage employees from voluntarily leaving the organisation.

### Career adaptability

The modern work landscape, marked by rapid changes in the digital age, is characterised by frequent job transitions, organisational shifts, and evolving career paths. This demands employees to enhance their agility and flexibility to adapt effectively (Rudolph et al., 2017; Du Plessis, 2021). In response to the unprecedented societal, economic, and technological forces reshaping the world of work in the digital era, Johnston (2019) defines career adaptability as the skill to positively regulate psychological and behavioural functions when confronted with new, changing, or uncertain circumstances. Career adaptability encompasses the intra-personal psychological capacities that serve as resources for managing one’s career and facilitating proactive adaptation to succeed in the rapidly evolving digital work environment, as noted by Hirschi et al. (2018).

In essence, Savickas (1997) career adaptability model empowers individuals to handle workplace uncertainties and changes, fostering optimism and resilience. It equips individuals with the skills and mindset necessary to navigate their careers effectively, ensuring relevance and resilience in the face of change. This adaptability empowers employees to seize new opportunities and maintain job satisfaction (Chen et al., 2020). In addition, high levels of career adaptability are associated with reduced voluntary turnover and a decreased intention to leave the organisation (Lee et al., 2021; Wang et al., 2021). When employees feel that they can adapt to new challenges, find opportunities for career growth within their current workplace, and continuously enhance their skills, they are less inclined to seek opportunities elsewhere. Career-adaptable employees tend to perceive their current organisation as a place where they can meet their long-term career aspirations, making them more likely to stay. Career adaptability therefore significantly contributes to employee retention (Lee et al., 2021; Sun et al., 2023). Employees who possess career adaptability are more engaged, committed, and satisfied with their jobs. They are better equipped to navigate potential job-related challenges and adapt to changes within the organisation. As a result, they are less likely to leave, ultimately contributing to improved employee retention rates.

### Integration

In conclusion, highly talented individuals are instrumental in the success of South African private sector organisations, making talent retention a top priority. To sustain success, organisations must holistically address talent planning, acquisition, attraction, leadership, development, deployment, rewards, engagement, and retention. Understanding the expectations of talented employees at both individual and collective levels is crucial. The challenge of retaining such individuals is widespread and could intensify as they become scarcer in the future. Organisations that do not strategically retain their top talent risk losing their competitive edge and overall productivity. Research has shown that these sought-after talents are often found in individuals who exhibit commitment, embeddedness, hardiness, adaptability, and resilience as discussed.

## Methods

### Participants

The study utilised convenience sampling, a method chosen to optimise the collection of usable questionnaires from employees in South African private sector organisations. Convenience sampling selects participants based on practical criteria, such as accessibility, availability, and willingness to participate (Venter et al., 2017). This approach is cost-effective and well-suited for quantitative studies (Stratton, 2021). The sampling frame specifically targeted full-time and part-time employees in South African private sector organisations, creating a sample with shared characteristics to facilitate meaningful conclusions about this group.

The research sample included 293 (*n* = 293) participants, primarily from global digital mindset human resource and financial service organisations, with a predominantly South African representation (70%). Additional participants came from Zimbabwe (15%) and Europe (15%). The gender distribution in the sample was nearly even, with 54% male and 46% female participants. In terms of racial demographics, the majority identified as belonging to Black racial groups (African, Indian, Asian, and Coloured), comprising 63% of the sample, while the remaining 37% identified as part of white racial groups. The composition of the sample is summarised in Table 1.

**Table 1:** Sample descriptives.

Age				
	Frequency	Percent	Valid percent	Cumulative percent
18–25 years	22	3.8	3.8	3.8
26–35 years	118	20.6	20.6	24.4
36–45 years	206	**35.9**	**35.9**	**60.3**
46–55 years	130	22.6	22.6	82.9
56–65 years	98	17.1	17.1	100.0
Total	574	100	100	
Gender				
Females	359	**62.5**	62.5	62.5
Males	215	37.5	37.5	100
Total	574	100.0	100.0	
Employment status				
Full time	471	**82.1**	84.7	84.7
Part time	85	14.8	15.3	100.0
Interns/Graduates	18	3.1	100.0	
Total	574	100.0		
Tenure				
Less than 5 years	207	36.1	36.1	36.1
6–10 years	293	**51.0**	51.0	87.1
11–15 years	58	10.1	10.1	97.2
More than 15 years	16	2.8	2.8	100.0
Total	574	100.0	100.0	
Job level				
Staff level	293	**51.0**	51.0	51.0
Supervisory level	91	15.9	15.9	66.9
Middle management level	74	12.9	12.9	79.8
Senior management level	59	10.3	10.3	90.1
Executive level	57	9.9	9.9	100.0
Total	574	100.00	100.0	

The bold values should be the highest values for that group.

### Measures

*The Organisational Commitment Scale* (Meyer and Allen, 1997), a 24-item scale, was applied to measure the dimensions of organisational commitment, which includes *affective commitment* (8 items), *continuous commitment* (8 items), and *normative commitment* (8 items). The respondents had to rate each item on a five-point Likert-type scale (1 = “strongly disagree”; 5 = “strongly agree”). Internal consistencies of the OCS dimensions vary.85 for affective commitment, 0.79 for continuance commitment, and.73 for normative commitment (Meyer and Allen, 1997). The validity of the construct is grounded in the expected connections between the three multifaceted constructs (Meyer and Allen, 1997).

*The Job Embeddedness Scale* (Mitchell et al., 2001), a 17-item scale was used to measure the three dimensions of job embeddedness: *fit*, *links*, and *sacrifice*. The items are rated on a six-point Likert-type scale (1 = “strongly disagree”; 6 = “strongly agree”). The internal consistencies of the JES dimension vary between 0.64 for organisational fit and 0.66 for organisational sacrifice or links, and the Cronbach Alpha values for all the other variables were higher than the recommended.70 (Mitchell et al., 2001).

The *Personal Views Survey* (PVS-III-R; Maddi et al., 2001), an 18-item multi-level scale, measures three features of hardiness: *the commitment* subscale (6 items), the *control* subscale (6 items), and the *challenge* subscale (6 items). The items are rated on a 4-point Likert-type scale (0 = “not true at all”; 3 = “completely true”). Kobasa (1982) found substantial test–retest correlations, with commitment at 0.69, control at 0.69, and challenge at 0.73, aligning with the findings in this study. Moreover, the subscales of the PVS-III-R demonstrated strong internal validity, with commitment at 0.85, control at 0.70, and challenge at 0.71, while the internal consistency reliability estimates (Cronbach Alpha) for all other variables remained consistent at.61.

The *Employee Resilience Scale* (EmpRes; Naswall et al., 2013), is a 9-item one-dimensional measure of employee resilience. The items are rated on a 7-point Likert-type scale (1 = “Almost never”; 7 = “Almost always”). The reliability of the revised scale was.91 (Naswall et al., 2015). Furthermore, the internal consistency reliability, calculated using Cronbach’s Alpha, for all the other variables surpassed the recommended threshold and fell within the range of 0.55 to 0.70.

The *Career Adaptability Inventory* (CAI: Savickas, 1997), a 35-item multi-level scale measures 4 dimensions of career adaptability: *concern* (8 items), *control* (9 items), *curiosity* (9 items), and *cooperation* (9 items). The items are rated on a 5-point Likert-type scale (1 = “Not strong”; 5 = “Strongest”). Concerning the internal consistency of the scale, the four subscales exhibited satisfactory values, ranging from 0.74 (Control) to 0.85 (Confidence). Moreover, the reliability estimates for all other variables remained consistently high at 0.88.

### Procedure

The researchers obtained ethical clearance from the research institution and gained permission from the participating organisations. Data collection was facilitated through an online survey. Initially, the CEOs of the organisations received an invitation letter containing a URL to the survey, which was disseminated to the HR Managers. These managers, in turn, shared the survey link with their respective employees.

The email communication included details about the research objectives, participant roles, estimated time for survey completion, the researcher’s contact information, privacy assurances, information use disclosure, and emphasised the voluntary nature of participation. Completion of the online survey served as participants’ informed consent. Throughout the data collection and analysis phases, strict measures were in place to preserve participant anonymity, with no collection of personal identifiers. Responses were coded to ensure confidentiality, and the collected questionnaires were securely stored through an automated transfer to a web-based platform.

### Data analysis

The data analysis was conducted using IBM Corp.’s SPSS Version 27 and SAS/STAT® software Version 9.4 M5^©^ (2017). To understand the relationships between the study variables, bivariate correlation analysis and stepwise regression analysis were performed.

Structural Equation Modelling (SEM) with maximum-likelihood (ML) estimation was employed to assess the fit between retention-related dispositions and resilience-related behavioural capabilities. The model’s goodness of fit was evaluated using various absolute goodness-of-fit indices, including the Chi-square test, Root Mean Square Error of Approximation (RMSEA), Standardised Mean Square Residual (SRMR), as well as the Comparative Fit Index (CFI) and Tucker-Lewis Index (TLI). Based on Garson’s (2008) guidelines, the structural model is considered to have a satisfactory fit to the measurement data when the following criteria are achieved: CFI and TLI values of 0.90 or greater, an RMSEA value of 0.08 or lower, and an SRMR value of 0.05 or lower.

## Results

### Descriptive statistics and correlations

Table 2 provides an overview of descriptive statistics, including means, standard deviations, and internal consistency reliabilities, along with correlations between study variables.

**Table 2:** Descriptive statistics and bi-variate correlations.

	Variable	α	CR	Mean (SD)	1	2	3	4	5	6	7	8	9	10	11	12	13	14	15	16	17	18
1	Affective Commitment	0.88	0.88	5.92 (0.95)	-																	
2	Normative Commitment	0.87	0.94	5.60 (0.96)	0.30**	-																
3	Continuance Commitment	0.87	0.87	5.52 (1.00)	0.40**	0.32**	-															
4	Overall OCS	0.94	0.96	5.69 (0.85)	0.39**	−0.30**	0.30**	-														
5	Fit	0.85	0.82	5.37 (0.64)	0.16**	0.25**	−0.32**	0.05	-													
6	Sacrifice/Links	0.96	0.95	4.08 (1.16)	0.08	−0.24**	0.07	0.06	0.43**	-												
7	Overall JES	0.94	0.92	4.62 (0.86)	−0.23**	0.34**	0.20**	0.12**	0.34**	−0.00	-											
8	Commitment	0.77	0.74	3.59 (0.59)	0.15**	0.04	−0.01	0.09*	−0.03	−0.07	0.27**	-										
9	Control	0.58	0.66	3.53 (0.35)	0.11**	0.05	0.03	0.11*	0.33**	−0.04	−0.13**	0.27**	-									
10	Challenge	0.83	0.84	3.11 (0.76)	0.17**	0.14**	0.04	0.33**	0.03	0.21**	0.05	0.47**	0.45**	-								
11	Overall PSV-R-III	0.85	0.84	3.37 (0.50)	0.11**	0.12**	−0.02	0.11*	0.15**	0.26**	−0.04	0.14**	0.39**	−0.26**	-							
12	Resilience	0.67	0.58	6.18 (0.43)	0.37**	0.04	0.04	0.19**	0.18**	−0.17**	0.34**	−0.15**	0.36**	0.02	−0.07	-						
13	Concern	0.84	0.85	4.47 (0.52)	0.40**	0.27**	0.10*	0.56**	−0.10**	0.32**	0.12**	0.31**	0.35**	−0.31**	0.08	−0.14**	-					
14	Control	0.81	0.80	3.94 (0.53)	0.57**	0.13**	0.42**	0.55**	0.27**	−0.35**	−0.03	0.22**	0.34**	−0.16**	−0.23**	0.32**	0.33**	-				
15	Curiosity	0.94	0.93	3.57 (0.83)	0.49**	0.47**	0.46**	0.91**	0.15**	0.04	−0.22**	−0.06	0.45**	0.16**	0.92**	0.35**	0.51**	0.58**	-			
16	Confidence	0.81	0.83	4.62 (0.54)	0.72**	0.54**	0.48**	0.87**	0.01	−0.10*	0.97**	0.49*	0.18**	−0.05	0.33**	0.13**	0.37**	0.21**	0.13**	-		
17	Cooperation	0.88	0.88	4.02 (0.50)	0.64**	0.54**	0.73**	0.87**	0.54**	−0.28**	0.72**	0.36*	0.11**	−0.31**	0.73**	0.37**	0.30**	0.30**	0.11**	0.39**	-	
18	Overall CAAS	0.92	0.95	3.97 (0.42)	0.22**	0.08	0.20**	0.47**	0.30**	−0.01	−0.31**	0.07	0.23**	0.28**	0.24**	0.36**	0.61**	0.48**	0.69**	0.65**	0.68**	-

**Cronbach alpha value.

The results revealed significant positive correlations among the three subscales of OCS, ranging from small to large practical effect sizes (*r* ≥ 0.03 ≤ 0.73; *p* ≤ 0.05; *p* ≤ 0.01). These subscales also correlated positively and significantly with overall JES, PVS-III-R, EmpRes, and CAS scales (*r* ≥ 0.10 ≤ 0.98; small to large practical effect size; *p* ≤ 0.05; *p* ≤ 0.01), demonstrating the construct validity of organisational commitment.

Concerning JES, significant positive correlations were observed between its two subscales, with small to large practical effect sizes (*r* ≥ 0.15 ≤ 0.54; *p* ≤ 0.05; *p* ≤ 0.01). These dimensions of JES also correlated positively and significantly with overall EmpRes and CAS scales (*r* ≥ 0.18 ≤ 0.32; small to medium practical effect size; *p* ≤ 0.05; *p* ≤ 0.01), confirming the construct validity of job embeddedness.

Regarding PVS-III-R, significant positive correlations existed among its three subscale dimensions, with small to medium practical effect sizes (*r* ≥ 0.18 ≤ 0.49; *p* ≤ 0.05; *p* ≤ 0.01). Commitment and control, the two subscale dimensions of PVS-III-R, also positively correlated with the overall CAS scale (*r* ≥ 0.22 ≤ 0.45; small to medium practical effect size; *p* ≤ 0.05; *p* ≤ 0.01), establishing the construct validity of hardiness.

As shown in Table 2, significant correlations were found between the one-dimensional measure of employee resilience and the EmpRes, with high reliability (*r* ≥ 0.13 ≤ 0.37; small to medium practical effect size; *p* ≤ 0.05; *p* ≤ 0.01). This measure also exhibited positive and significant correlations with CAS’s four subscales (concern, control, curiosity, and cooperation; *r* ≥ 0.13 ≤ 0.36; small to medium practical effect size; *p* ≤ 0.05; *p* ≤ 0.01) and the overall CAS scale (*r* ≥ 0.37; medium practical effect size; *p* ≤ 0.05), indicating the construct validity of resilience.

In summary, the findings indicated positive and significant correlation variables related to OCS, JES, PVS-III-R, EmpRes, and CAS, with scores ranging from small to large practical effect sizes.

### Stepwise regressions analysis

Model 1, which considered socio-demographic variables alone, yielded the following results: *F* = 9.99; *p* = 0.000; Adjusted *R^2^* = 0.89 (small to large practical effect size). Model 2 incorporated socio-demographic variables, job embeddedness, and hardiness, with these results: *F* = 20.83; *p* = 0.000; Adjusted *R^2^* = 0.15 (small practical effect size). Model 3 included socio-demographics, job embeddedness, hardiness, and organisational commitment as independent variables, and its results were as follows: *F* = 1.14; *p* = 0.000; Adjusted R^2^ = 0.15 (small practical effect size). Model 4 encompassed socio-demographics, the independent variables (organisational commitment, job embeddedness, and hardiness), and their interactions in predicting the dependent variables (resilience and career adaptability). The results were as follows: *F* = 26.31; *p* = 0.000; Adjusted *R^2^* = 0.22 (small practical effect size).

In Model 2, job embeddedness played the most significant role in explaining the variance in resilience and career adaptability (*β* = 0.15; t-value = 6.26), while hardiness contributed to a lesser extent in explaining the variance in resilience (*β* = −0.03; t-value = −0.86). Job embeddedness also predicted a significant and positive relationship between the dependent variables (resilience and career adaptability) and socio-demographic variables.

In Model 3, job embeddedness was the most substantial contributor in explaining the variance in resilience and career adaptability (*β* = 0.17; t-value = 5.88). Organisational commitment (*β* = −0.03; t-value = −1.07) and hardiness (*β* = −0.02; t-value = −0.43) had a comparatively smaller impact on explaining the variance in the resilience and career adaptability constructs. Additionally, all tolerance scores were high (OC = 0.54; JE = 0.49; hardiness = 0.72).

In Model 4, job embeddedness continued to be the most significant factor in explaining the variance in resilience and career adaptability (*β* = 0.18; t-value = 5.97). Organisational commitment (*β* = −0.05; t-value = −1.82) and hardiness (*β* = −0.04; t-value = −1.01) had a relatively minor role in explaining the variance in these constructs. There was a positive interaction effect between cJE*cOC (*β* = 0.08; t-value = 4.32) in predicting resilience and career adaptability, and conversely, a negative interaction between cHardiness*cOC (*β* = −28; t-value = −6.79). Furthermore, the tolerance scores for all independent concepts and socio-demographic variables were high, except for race = 0.14. Thus, the results indicated that all socio-demographic variables (except race and employment status) positively and significantly predicted the relationship between organisational commitment, job embeddedness, hardiness, resilience, and career adaptability (Table 3).

**Table 3:** Final step: stepwise regression analysis.

Variables	Estimate(β)	Standard error	t-value	*p*	Tolerance
Constant	6.09	0.05	14.78	0.00	–
Age	0.01	0.03	0.23	0.82	0.68
Gender	−0.01	0.04	−0.36	0.72	0.87
Race	−0.07	0.04	−0.1.60	0.11	0.89
Marital status	0.15	0.04	4.29	0.00	0.93
Job level	0.16	0.04	3.72	0.00	0.76
Employment status	−0.14	0.05	−0.2.88	0.00	0.92
Model 1 summary					
*F*	9.99				
*P*	0.000				
Adjusted R^2^	0.89				
Constant	5.58	0.17	32.80	0.00	–
Age	−0.03	0.03	−0.1.05	0.29	0.62
Gender	0.02	0.04	0.06	0.95	0.86
Race	−0.08	0.04	−1.78	0.77	0.87
Marital status	−0.12	0.04	3.26	0.01	0.88
Job level	0.06	0.04	1.35	0.77	0.65
Employment status	−0.10	0.05	−2.02	0.04	0.65
Job embeddedness	0.06	0.04	1.35	0.18	0.68
Hardiness	−0.03	0.04	−0.86	0.38	0.81
Model 2 summary					
*F*	20.83				
*P*	0.000				
Adjusted R^2^	0.15				
Constant	5.61	0.17	32.39	0.00	–
Age	−0.03	0.03	−0.85	0.40	0.60
Gender	2.5	0.04	0.00	0.99	0.86
Race	−0.08	0.04	−0.1.80	0.07	0.87
Marital status	0.11	0.04	3.21	0.00	0.88
Job level	0.06	0.04	1.37	0.17	0.65
Employment status	−0.10	0.05	−2.08	0.04	0.90
Job embeddedness	0.16	0.03	5.88	0.00	0.49
Hardiness	−0.02	0.04	−0.43	0.67	0.71
Organisational commitment	−0.03	0.03	−1.07	0.29	0.54
Model 3 summary					
*F*	1.14				
*P*	0.29				
Adjusted R^2^	0.15				
Constant	5.72	0.19	30.34	0.00	–
Age	−0.01	0.03	0.17	0.87	0.57
Gender	0.01	0.04	0.24	0.81	0.85
Race	−0.06	0.04	−1.49	0.14	−0.14
Marital status	0.09	0.03	2.77	0.01	0.87
Job level	0.03	0.04	0.79	0.43	0.62
Employment status	−0.12	0.05	−2.53	0.01	0.89
Job embeddedness	0.18	0.03	5.97	0.00	0.41
Organisational commitment	−0.05	0.03	−1.82	0.07	0.53
cJE*cOC	0.08	0.02	4.32	0.00	0.71
cHardiness*cOC	−0.28	0.04	−6.79	0.00	0.77
Model 4 summary					
*F*	26.31				
*P*	0.000				
Adjusted R^2^	0.22				

*N* = 574; OC; organisational commitment; dependent variables: Resilience and career adaptability; cJE*cOC, cHardiness*cOC: interaction effect between the concepts; c: means that the variables are mean centred.

In summary, the findings revealed that the independent factors (organisational commitment, job embeddedness, and hardiness) and six socio-demographic variables (age, gender, race, marital status, job level, and employment status) significantly predicted both resilience and career adaptability. Specifically, all dimensions of organisational commitment (affective commitment, continuance commitment, and normative commitment) positively and significantly predicted resilience and career adaptability. In the case of job embeddedness, both its dimensions (fit and sacrifices/links) played significant roles in explaining resilience and career adaptability. Conversely, the dimensions of PVS-III-R had a negative relationship with the dependent variables (resilience and career adaptability).

### Structural equation model

Expanding upon the correlation analysis, structural equation modelling was employed to assess the overall fit of the structural model. The fit statistics indicated that the model tested fits the data satisfactorily, and thus, the model is acceptable: Chi-square (30.90), RMSEA (Root Mean Square Error of Approximation) = 0.060, SRMR (Standardised Root Mean Square Residual) = 0.13, CFI (Comparative Fit Index) = 0.82, TLI (Tucker-Lewis Index) = 0.81. The goodness-of-fit statistics further validate that the attribute of agility and adaptability significantly predicts the construct of value-oriented psychological contract (*β* = 0.60; *p* = 0.000; Table 4).

**Table 4:** Model fit statistics: competing structural models.

Model	Chi-square	*p*	RMSEA	SRMR	CFI	TLI	AIC	BIC
1	30.90	0.000	0.06	0.13	0.82	0.81	95110.33	96115.79
2	0.33	0.000	0.00	0.00	1.00	1.00	3782.85	3952.60

Considering the model’s goodness of fit, the proposed resilience framework is recommended for private-sector organisations in South Africa.

## Discussion

In a world grappling with a talent shortage, organisations focus on retaining top-performing employees for their competitive edge. This challenge is amplified in Africa and more specifically South African private-sector organisations. While various retention strategies have been employed, retaining top talent goes beyond rewards; it entails creating satisfying work environments and aligning employee expectations with organisational goals. To address this, South African private sector organisations must move beyond traditional retention strategies by investing in organisational commitment, job embeddedness, hardiness, resilience, and career adaptability. This study explores the relationship dynamics between retention-related dispositions and resilience-related behaviours, offering insights into talent retention in South African private sector organisations and addressing research gaps.

### Summary of findings

The analysis of construct descriptives highlights participants’ profound emotional attachment to the organisation, extending well beyond their job roles. This deep connection fosters a strong sense of belonging and loyalty, which deeply influences their behaviour and performance. Furthermore, above-average scores on the Job Embeddedness Scale (JES) underscore participants’ strong alignment with their jobs, community and organisation. While the Personal Views Survey (PVS-III-R) shows moderate satisfaction with commitment and control, there is room for improvement in addressing the marginal satisfaction with challenge. Employees’ desire for more growth and challenges in their roles can enhance job satisfaction and overall well-being. Moreover, positive scores on the Employee Resilience Scale (EmpRes) reveal participants’ resilience, empowering them to adapt and recover effectively from challenges, contributing to their ongoing engagement and productivity. Lastly, the Career Adaptability Scale (CAS) reflects average scores across most subscales, with a distinct high confidence in participants regarding their careers. This self-assuredness can lead to increased career mobility, encouraging employees to explore new opportunities, embrace challenges, and take an active role in their career development.

The results further show that positive perceptions of organisational commitment, job embeddedness, and hardiness are the most influential factors in predicting resilience-related behavioural capacities and career adaptability. A strong sense of organisational commitment fosters a positive and supportive work environment, nurturing a psychological attachment to the organisation (Moeng et al., 2023). This, in turn, leads to increased job satisfaction and influences various aspects of employee behaviour and experiences. These findings align with research conducted by Sharma et al. (2021) and Moeng et al. (2023), supporting the idea that employees with higher organisational commitment are better equipped to cope with stress and adversity.

Moreover, employees who feel deeply embedded in their roles and organisation experience a profound sense of belonging, which enhances their job satisfaction. Mitchell and Lee (2001) define “fit” as the alignment between an individual and the organisation’s culture. This alignment fosters a sense of belonging and is associated with increased employee retention (Pillay, 2020). High levels of job embeddedness are also linked to greater job satisfaction and reduced turnover intentions (Potgieter and Ferreira, 2018).

In addition to organisational commitment and job embeddedness, hardiness plays a pivotal role in equipping employees with the psychological strength to overcome adversity and facilitate career growth. High-hardi individuals tend to fully commit to their tasks, believe in their ability to influence their life events and view change as an opportunity for personal development and growth (Ferreira, 2012). This perspective on challenge and change positively influences affective and continuance commitment. Employees who embrace challenging experiences as opportunities for personal growth are more likely to remain committed to the organisation. Furthermore, these employees are often motivated to act as change agents in their environment and are less inclined to leave the organisation, as they perceive the costs of departure to be prohibitively high.

In summary, high levels of organisational commitment, job embeddedness, and hardiness have been shown to reduce turnover. When employees are committed to their organisation and deeply embedded in their roles, they are more likely to stay, fostering a stable workforce. Hardiness contributes to this by enabling employees to persevere in the face of challenges, making them more committed and less inclined to seek opportunities elsewhere.

**Figure 1:**
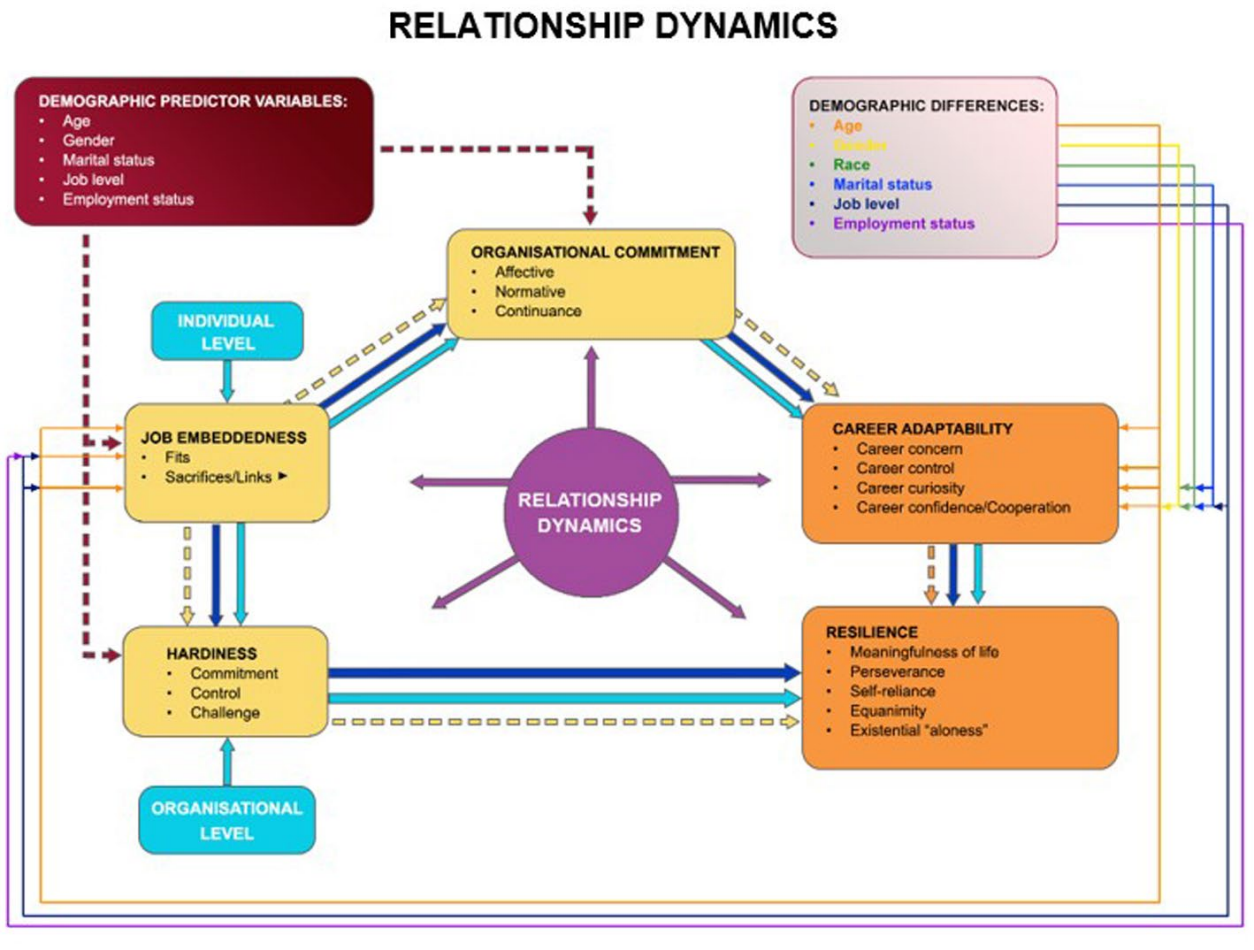
Empirically manifested resilience framework for South African private sector organisations. Source: authors own work.

The cumulative impact of these constructs on talent retention is substantial, as they collectively form comprehensive and effective strategies for retaining highly talented individuals. In conclusion, this discussion informs the development of an integrated theoretical resilience framework for South African private sector organisations, emphasising the significance of considering these constructs in the formulation of effective retention strategies. Figure 1 below visually represents the empirically established resilience framework. This distinctive framework can serve as a valuable guide when designing talent retention management strategies and practices tailored to South African private sector organisations.

### Theoretical implications

This study developed a theoretical resilience framework to inform talent retention practices in South African private sector organisations. It emphasised the dynamic relationships between retention-related dispositions (organisational commitment, job embeddedness, and hardiness) and resilience-related behavioural capacities (resilience and career adaptability), and how these variables relate to contemporary talent retention initiatives. The insights gained from this study, particularly regarding the interplay between the identified variables, have several practical implications for South African private-sector organisations.

### Practical implications

The proposed resilience framework offers a holistic approach to talent retention in private sector organisations. It aids in designing unique strategies to keep top talent and stay competitive. The implications for HR and management are as follows:

First, prioritise strong relationships between employees and their organisations. Recognising the importance of organisational commitment in reducing employee turnover, management should strive to create a positive work environment that fosters a sense of belonging, autonomy and loyalty. This can be achieved through employee engagement programmes, job design and crafting, recognition, rewards, and career development (Dutta et al., 2023; Jay, 2023).

Second, promote job embeddedness by creating a sense of community, offering flexible work arrangements, and providing support and resources such as employee support groups and mentorship programmes. In addition, management should ensure that their employees are satisfied with their compensation, training and development and career development opportunities (Shibiti, 2019).

Third, identify and nurture employees with hardiness, a valuable trait. Develop hardiness in employees by providing training on stress management, resilience building, promote a growth mindset, and create a supportive and collaborative work environment.

Fourth, emphasise the importance of resilience, particularly in high-positive affectivity individuals, as a psychological resource against job demands. Dedicate resources to enhance coping skills and well-being through initiatives like stress management, mental health support and maintaining a healthy work-life balance (Rodríguez-Sánchez, 2021).

Lastly, support employees in developing and enhancing their career adaptability through training, mentorship, and career development initiatives (Gama Jobs, 2023). Encourage a culture of continuous learning and adaptability within the organisation to ensure employees are better prepared to navigate transitions and embrace new opportunities.

These suggestions offer management the means to enhance talent retention, reduce turnover intention, and create a more engaged and adaptable workforce. Prioritising organisational commitment, promoting job embeddedness, nurturing hardiness, emphasising resilience, and supporting career adaptability all contribute to improved employee well-being and a competitive advantage in the dynamic business landscape.

### Limitations and future research

The study’s conclusions need to be considered considering the limitations stemming from its cross-sectional research design. To enhance the generalisability of the findings, future research should aim to replicate the study in a more extensive range of industry contexts with larger participant samples. Conducting longitudinal studies may also prove beneficial in investigating agile adaptable attributes and value-oriented commitments across various settings and diverse population groups. Future research could further explore the moderating role of socio-demographic variables as these variables can significantly influence how retention practices are perceived and applied.

## Conclusion

In a world facing a talent shortage, South African private sector organisations grapple with retaining skilled employees (Van Zyl et al., 2017). This study underscores the need for a holistic approach to talent retention by accentuating the interconnected nature between various retention-related attributes and resilience-related behavioural capacities. The study found that positive perceptions of organisational commitment, job embeddedness, and hardiness were influential in predicting resilience and career adaptability, leading to increased job satisfaction and reduced turnover intentions.

In conclusion, this study offers a theoretical framework for talent retention in South African private-sector organisations. Prioritising them enhances talent retention and fosters an engaged and adaptable workforce. Nonetheless, the study has limitations, and future research should explore these constructs in different industries, larger samples, and longitudinal studies. Examining the impact of socio-demographic variables on talent retention is also crucial.

## Data availability statement

The original contributions presented in the study are included in the article/supplementary material, further inquiries can be directed to the corresponding author.

## Ethics statement

The studies involving humans were approved by Ethical clearance to conduct the research was obtained from the University of South Africa (ERC Ref#: 2019_CEMS/HRM_010). Participants’ anonymity, privacy, and voluntary participation were respected. The studies were conducted in accordance with the local legislation and institutional requirements. The participants provided their written informed consent to participate in this study.

## Author contributions

NF: Conceptualization, Supervision, Writing – original draft. EM: Conceptualization, Data curation, Formal analysis, Writing – review & editing. MP: Writing – review & editing.
